# Sector analysis reveals patterns of cambium differentiation in poplar stems

**DOI:** 10.1093/jxb/ery230

**Published:** 2018-06-20

**Authors:** Gerd Bossinger, Antanas V Spokevicius

**Affiliations:** The University of Melbourne, School of Ecosystem and Forest Sciences, Creswick, Victoria, Australia

**Keywords:** Cambium initials, cell fate, poplar, stem cells, wood formation, xylogenesis

## Abstract

We used sector analysis to study cambium development and dynamics and to test whether fundamental developmental and functional differences exist between cambial initials as true ‘stem cells’ and more differentiated mother cells. In many higher plants, a cylindrical lateral meristem, the vascular cambium, forms along the plant axis. Most notably in stems of perennial tree species, this meristem gives rise to xylem (wood) towards the inside of the trunk and phloem (bark) towards the outside. As such, the vascular cambium is responsible for the production of most of the planet’s forest biomass, significantly contributing to the global carbon cycle. Using the bacterial *uidA* reporter gene in *Agrobacterium*-based *in vivo* stem transformation experiments in poplar trees, we created 379 cambium sectors that originated from the transformation of individual cells. Results from our analysis of sector frequency and patterns are consistent with the poplar cambium featuring a single layer of true cambial initials (being able to divide both anti- and periclinally). We show that initials are frequently lost from the cambium, that such cell loss rarely occurs at mother cell level, that phloem and xylem differentiation are controlled independently, and that the frequency of mother cell replenishment is not pre-determined.

## Teaser

Sector analysis in poplar tree stems was used to shed light on cell identity, cell fate, and patterns of development within the vascular cambium.

## Introduction

The vascular cambium is a meristem that during secondary growth of perennial woody plant species produces wood (xylem) towards the inside of the stem and bark (phloem) towards the outside. Cambial differentiation, particularly during secondary growth, is arguably one of the planet’s most important biological processes as, with an estimated global forest growing stock of 530.5 billion m^3^ (Köhl *et al.*, 2015), it is responsible for the production of much of the world’s biomass. Using forest inventory data and long-term ecosystem studies, the total carbon sink in the world’s established forests has been calculated to be 2.4 ± 0.4 billion tons of carbon per year (estimated for the period 1990–2007 by Pan *et al.*, 2011), highlighting the importance of the cambium and the forest biomass it produces as a contribution to the global carbon cycle.

The cambium exists as a tier of meristematic cells found in a tree stem between xylem and phloem tissues. Cambial cells undergo mostly periclinal cell divisions that occur parallel to the closest organ surface and are responsible for the addition of cells along a radial file, but also anticlinal divisions that occur perpendicular to the closest organ surface and are responsible for the addition of new cells to the initiating layer and the addition of new radial files (Barlow *et al.*, 2002). The cambium is comprised of distinct zones, the division zone, where all cell divisions occur, and the maturation or differentiation zones, where cells undergo a variety of processes on their way to occupying their final position in the plant stem. The division zone is generally comprised of cambial initials enclosed on both radial walls by xylem and phloem mother cells (from here on referred to as XMCs and PMCs, respectively).

Over recent years, poplars have consolidated their role as a preferred model system for woody plant species, and our understanding of the molecular control of cambial cell proliferation and differentiation during later stages of secondary growth and wood formation has seen significant advances (Fisher and Turner, 2007; Hirakawa *et al.*, 2010; Etchells *et al.*, 2013, 2015; Kondo *et al.*, 2014; Zhang *et al.*, 2015). In comparison, our current knowledge of cambial cell identity and cell fate has advanced little from early concepts based on histological observations that in some cases are more than a century old (Sanio, 1873; Raatz, 1892).

So, what do we know about cambial initials and their fate? The term ‘cambial initial’ refers to two distinct cell types, fusiform and ray initials, which are responsible for longitudinally or transversely aligned cambial derivatives, respectively. Fusiform initials are responsible for the formation of all longitudinally aligned cells including vessels, fibres, and sieve tube elements, and are large (compared to ray initials), longitudinally elongated, and highly vacuolated (Catesson, 1980). Ray initials, on the other hand, will give rise to transversely aligned cells, primarily ray parenchyma cells, and occur as small to large scattered aggregations within the cambial zone (Bailey, 1923; Lev-Yadun and Aloni, 1995). Mother cells differ from initials as conceptually they are at a more advanced state of differentiation, being destined to become either xylem or phloem elements, whereas initials retain the ability to produce both (Catesson and Lachaud, 1993; Larson, 1994; Lachaud *et al.*, 1999). In the differentiation zone, cells, depending on their fate, undergo enlargement, elongation, cell wall deposition, lignification, and programmed cell death (PCD).

The existence of one versus multiple layers of true initials within the cambial zone remains a subject for debate due to difficulties associated with studying this region (Butterfield, 1975; Schmid, 1976; Larson, 1994, and many others) and the fact that true initials are largely indistinguishable from XMCs and PMCs. Using current anatomical, cytological, biochemical, or molecular techniques, the cambium is not accessible for *in planta* observation without disrupting normal developmental processes, and there are many difficulties associated with preserving and fixing cambial tissue for histological assessment (Catesson, 1974, 1980; Schmid, 1976; Larson, 1994; Barlow *et al.*, 2002).

Historically, two opposing models were presented by Sanio (1873), who proposed a uniseriate model involving one initiating cell within a radial file, and Raatz (1892), who proposed a multiseriate model involving several initiating cells in a radial file. In Sanio’s uniseriate model, a single initial gives rise to either a PMC or an XMC which divides again forming two daughter cells. Since these divisions result in the production of four cells, this structure has since been referred to as Sanio’s four. Similarly, Raatz in his multiseriate model proposes a progression of mother and daughter cells, but initiation can occur from any number of cells in a radial file rather than from a single defined cell.

Genetically tagging individual cells within the cambial zone and following their respective fate might shed light on predominant division patterns and pre-determined cell identities. Such analyses of genetic mosaics have greatly aided our understanding of fundamental aspects of pattern formation in animal (Nesbitt and Gartler, 1971) and plant systems (Spena and Salamini, 1995; Szymkowiak and Sussex, 1996; Bossinger and Spokevicius, 2011). For example, genetic tagging using the β-glucuronidase (GUS) reporter gene has been a valuable tool to visualize the clonal relationships of cells and to understand the formation of developmental patterns in plants (Scheres *et al.*, 1994; Bossinger and Smyth, 1996; Kilby *et al.*, 2000; Saulsberry *et al.*, 2002). However, these studies have focused solely on primary tissues. In earlier work, we started extending such investigations to secondary stems (Spokevicius *et al.*, 2006; Van Beveren *et al.*, 2006), merely scratching the surface of our understanding of secondary stem developmental patterning and growth dynamics, with many questions remaining. In this study, we go further by reporting on how we used transgenic approaches to label specific cell types in secondary stems of poplar trees in an effort to unravel the structure of the meristematic layer of the cambium as well as other aspects of developmental patterning and meristematic activity within this ecologically and economically important plant meristem.

## Materials and methods

### Plant material

Sixteen clonal *Populus alba* ‘*pyramidalis*’ L. plants growing at the University of Melbourne’s Creswick Campus, Victoria, Australia, were sourced from cuttings treated with rooting hormone powder (Yates rooting powder, Australia) in cutting beds. Plantlets were grown under controlled glasshouse conditions, where temperatures ranged from 14 °C to 17 °C at night and from 21 °C to 25 °C during the day, for several months prior to experimentation. Extra light was provided during winter months to achieve >12 h photoperiods. All plantlets were watered regularly and fertilized with slow-release fertilizer (Osmocote, Yates, Australia) every 6 months. At 6 months of age, 10 plantlets were selected and subjected to experimentation as described below. Plantlet height and diameter at 10 cm stem height were measured at the beginning and completion of our experiments.

### Cambial transformation using induced somatic sector analysis (ISSA)

Prior to transformation of cambial tissue, the pCAMBIA 1305.1 vector containing the GUSPlus™ reporter gene driven by the *Cauliflower mosaic virus* (CaMV) 35S promoter (http://www.cambia.org) was transformed into *Agrobacterium tumefaciens* strain AGL-1 (Lazo *et al.*, 1991). For inoculation, *A. tumefaciens* was grown in LB with the appropriate antibiotics to an OD_600_ of 0.4–0.6 and centrifuged. After decanting liquid, bacteria were immediately suspended in 1 ml of cooled Murashige and Skoog (MS) medium and stored on ice until needed. ISSA transformation was performed as described in Spokevicius *et al.* (2016). In brief, a series of cambium windows or upward bark flaps were created on the stems of young poplar trees by making two parallel incisions of 20 mm length, 5 mm apart and connected on their lower end by a horizontal cut through the bark. Bark flaps were then peeled upward exposing the developing xylem. For inoculation, 5–10 μl of *Agrobacterium* solution was added to each window, bark flaps were then pushed back into place, and immediately tightly wrapped with parafilm (Bemis NA, USA). In total, 60 cambium windows were created across 16 plants.

Approximately 4.5 months after transformation, stems were harvested and stem sections at each window were excised and further sectioned into disks of between 0.5 mm and 1 mm in thickness (Spokevicius *et al.*, 2016).

### Sample assessment

**Fig. 1.**
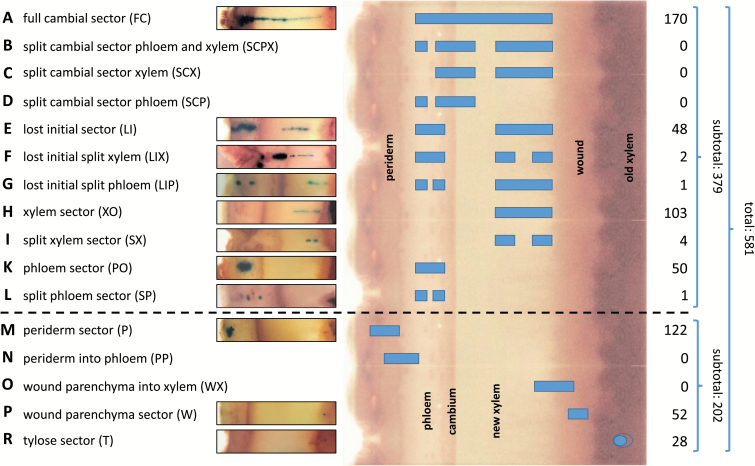
Transgenic sector patterns and number of observed cases for each sector type. Following ISSA *in vivo* transformation of exposed cambial tissue in poplar tree stems using a CaMV 35S GUSPlus™ construct, further growth of individual transformed cells led to the creation of individual transformed sector types and patterns. Observed and theoretical patterns and sector types are listed (A–R) together with examples of cross-sections of observed cases. In the centre, sectors and sector patterns are superimposed on a stylized cross-section of a poplar stem segment showing bark (periderm and phloem) to the left and newly formed wood (xylem), wound parenchyma, and old xylem (which had formed prior to the creation of a bark flap) to the right. On the far right, numbers of observed cases are provided for each sector type. A dashed line delineates those sectors that are deemed to have originated from transformed cells giving rise to cambial initials or mother cells from those that did not contribute to cambium formation.

Samples were screened for GUS staining using a stereomicroscope (Leica MZ FLIII, Wetzlar, Germany), and measurements of the quantity and quality of new radial growth in each cambial window were taken. Specifically, measurements were taken of total new radial growth (including wound parenchyma and xylem) and new radial xylem only growth. GUS-positive tissues and cells were initially classified according to sector categories outlined in Spokevicius *et al.* (2016) (Fig. 1) with additional sector subtypes defined as part of this analysis. Sectors that appeared in regions with distorted wood formation and therefore did not represent ‘normal’ developmental patterns were not included in our assessment.

For more detailed visualization, nine stem sections containing stably transformed cambial sectors were fixed in 5% glutaraldehyde (ProSciTech) in 100 mM phosphate buffer for 2–3 d and dehydrated in an ethanol/0.1 M phosphate buffer dilution series (50, 70, and 100% ethanol, 2–5 d in each). Samples were infused with LR white acrylic resin (ProSciTech) using an LR white/ethanol dilution series (50, 70, and 100%, 2–5 d in each) and polymerized at 65 °C for 2 d prior to sectioning. Sections 2–5 µm thick were cut using an automatic microtome (Reichert-Jung 1140/autocut), mounted on glass slides with distilled H_2_O, and allowed to dry overnight. The following day, sections were stained with 1% safranin solution, mounted in Entellan synthetic resin (Merck), and allowed to set under light pressure. Finally, sections were analysed microscopically using an Olympus BH-2 light microscope.

## Results

### Plant growth

During the 4.5 months of experimentation, plants grew on average 123.2 (±10.5) cm in height and 6.5 (±0.6) mm in diameter (at 10 cm stem height). Total radial growth in cambial windows was on average 3.2 (±0.1) mm, with an average of between 96% (minimum) and 98% (maximum) of this growth consisting of new xylem tissue excluding wound parenchyma.

### Wound response and establishment of a new cambium

**Fig. 2.**
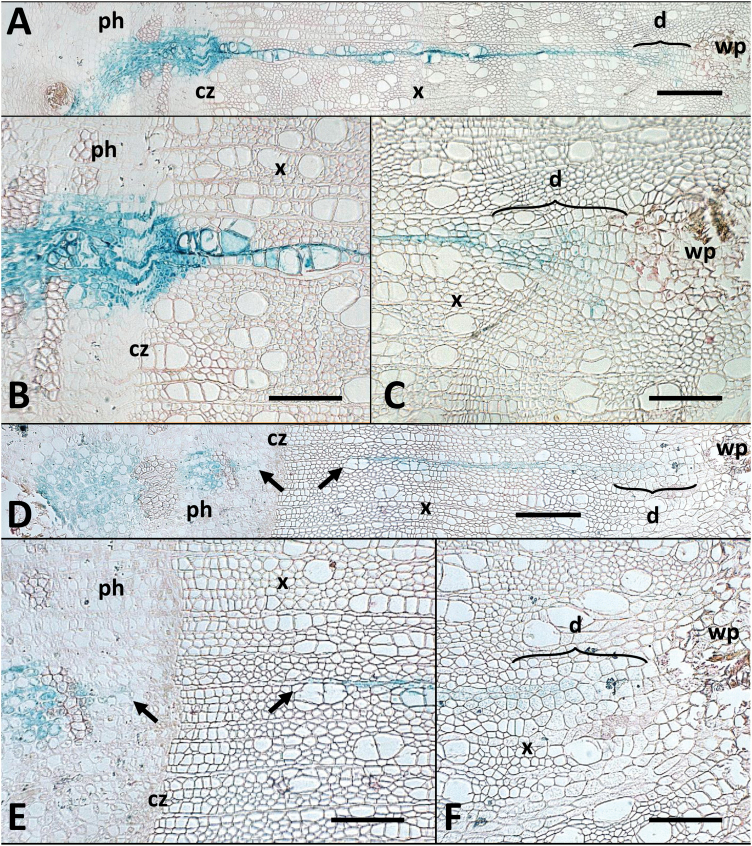
Micrographs of cross-sections showing histological detail of a continuous ‘full’ cambial sector and a split ‘lost initial’ sector. Micrographs show a continuous ‘full’ cambial sector (p46-3 in Supplementary Table S1; A–C) extending from the wound parenchyma (wp), through the xylem (x) and the cambial zone (cz) into the phloem (ph); and a split ‘lost initial’ sector (p33-11 in Supplementary Table S1; D–F) in which the phloem and xylem portions are radially aligned but separated across the cambial zone (cz). The gap across the cambial zone is indicated by arrows in (D) and (E). The dedifferentiation zone (d) is marked in (A), (C), (D), and (F). Scale bars: (A, D)=400 μm; (B, C, E, F)=200 μm.

Wound response was essentially as described earlier (Van Beveren *et al.*, 2006) with wound parenchyma developing between pre-existing wood and newly developing xylem. A new cambium was established after a small number of cell cycles most probably from pre-existing cambial cells that upon peeling remained with the bark flap (including initials, mother cells, and potentially developing phloem and xylem cells that underwent dedifferentiation). This is demonstrated by the presence of parenchyma cells in a short dedifferentiation zone (d in Fig. 2) between the wound parenchyma proper (wp in Fig. 2) and newly formed xylem. The latter in turn is characterized by the presence of vessel elements and fibre cells that have lost their cellular content (Fig. 2C, F). For the purpose of sector classification below, the dedifferentiation zone is included under ‘newly formed xylem’.

### Sector patterns and classification

GUS staining in transformed sectors was observed in all cell types and tissues, but gradually decreased as cellular content was lost during the maturation of fibre cells and vessel elements. With the exception of tylose sectors, no single cell staining was observed, confirming that *Agrobacterium* did not persist in cambial windows past the initial inoculation.

In total, 581 independently transformed tissue and cell (tylose) sectors were identified, classified, and selected for further analysis across the 60 windows assessed (Fig. 1). A comprehensive list of samples and identified sectors is presented in Supplementary Table S1 at *JXB* online.

A total of 379 or 65% of all sectors were deemed to have originated from cambial initials or mother cells, 122 sectors (21% of all sectors) were identified as periderm sectors (P; Fig. 1M), 52 (9%) as wound parenchyma sectors (W; Fig. 1P), and 28 (5%) as tylose sectors (T; Fig. 1R). Of the 379 sectors originating from initials or mother cells, 51 (13%) were characterized as ‘phloem only’ sectors (PO; Fig. 1K, L), 221 (58%) as ‘cambial’ sectors (C; Fig. 1A, E–G), and 107 (28%) as ‘xylem only’ sectors (XO; Fig. 1H, I). A total of 170 (77%) of ‘cambial’ sectors were identified as being ‘full’ (FC; Fig. 1A) and 51 (23%) as ‘lost initial’ (LI; Fig. 1E–G) cambial sector types. These sector types represent by far the predominant cell division patterns in the cambial region.

Only in eight cases (2%) did sectors deemed to have originated from cambial initials or mother cells show modifications to these patterns. These cases were classified into four sector subtypes including two ‘lost initial and split xylem’ (LIX), where two xylem sectors were aligned radially with each other and with a phloem sector (0.5%; Fig. 1F); one ‘lost initial and split phloem’ (LIP), where two phloem sectors were aligned radially with each other and with a xylem sector (0.3%; Fig. 1G); four ‘split xylem’ (SX), where two xylem sectors were aligned radially (1%; Fig. 1I); and one case of an additional phloem sector type ‘split phloem’ (SP), where two phloem sectors were radially aligned (0.3%; Fig. 1L).

No sectors were identified that crossed the periderm/phloem (PP) or the wound site/newly formed xylem (WX) tissue zones (Fig. 1N, O) and neither did we find any sectors that crossed the cambium into phloem and xylem and radially aligned with separate phloem or xylem sectors (SCPX, SCX, or SCP in Fig. 1B–D).

In summary, the vast majority of sectors that presumably derived from cambial activity (379), with very few exceptions, were full cambial sectors (170) followed by xylem sectors (103), phloem sectors (50), and lost initial sectors (48). No sectors were found that crossed the periderm/phloem or wound/newly formed xylem boundaries (Fig. 1). These sectors can therefore be regarded as reflecting predominant developmental patterns of cambium differentiation.

### Macroscopic assessment of ‘lost initial’, ‘phloem only’, and ‘xylem only sectors’

**Fig. 3.**
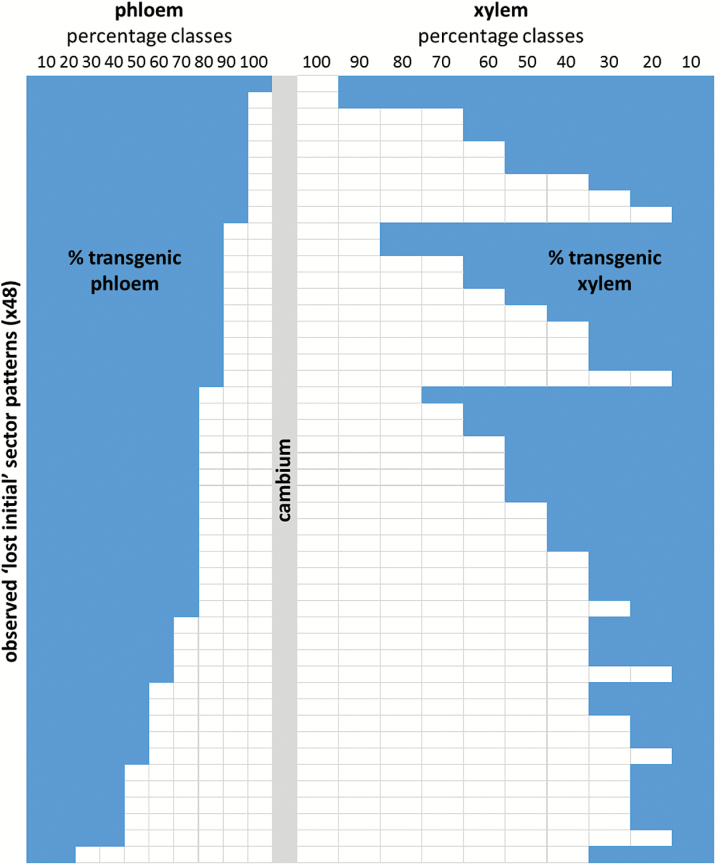
Pattern distribution in 48 ‘lost initial’ sectors. Absolute values for transgenic sector coverage were grouped into 10% increment classes and plotted according to phloem sector size in decreasing order (top to bottom), with relative phloem coverage to the left and xylem coverage to the right. While there is a general trend in ‘lost initial’ sectors that smaller phloem portions align with smaller xylem portions, this representation of our data shows that large and medium size phloem portions are equally likely to align with xylem portions of any size, indicating that phloem and xylem differentiation are not synchronized and are therefore likely to be controlled independently.

Relative contributions of transgenic (TG) tissue to both phloem and xylem were measured for 48 ‘lost initial’ sectors and expressed as %TG (Fig. 3). Absolute values were grouped into 10% increment classes and plotted according to phloem sector size in decreasing order (top to bottom). While there is a general trend that smaller phloem sectors align with smaller xylem sectors, it is also apparent that large and medium size phloem sectors are equally likely to align with xylem sectors of any size.

**Fig. 4.**
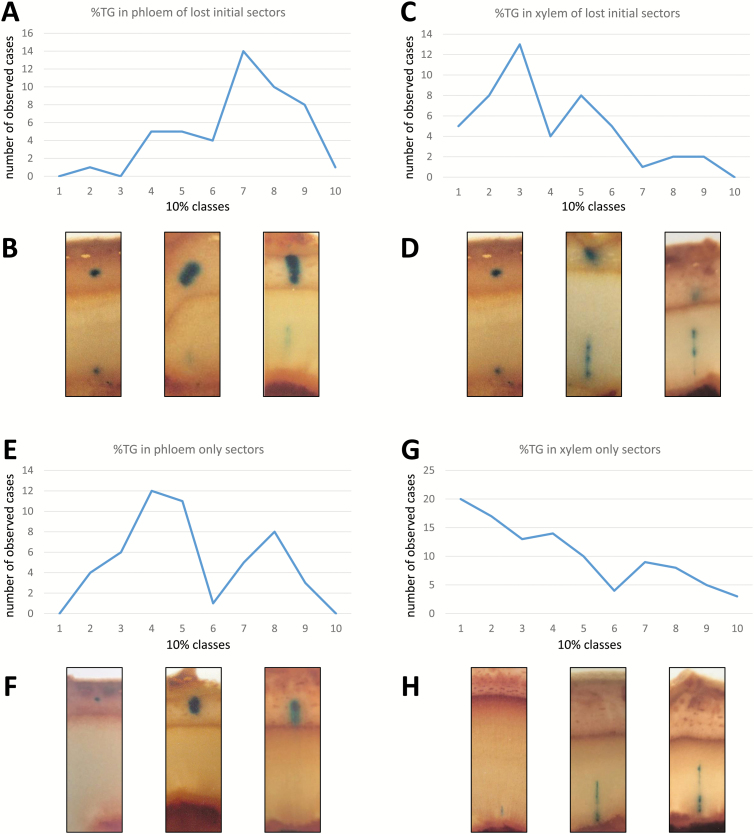
Patterns of replacement and replenishment of cambial initials and mother cells. Number of transgenic sectors in 10 percentage classes representing patterns of replacement of cambial initials (A, B) and replenishment of mother cells (E, G), respectively. Examples of sector cross-sections in corresponding size classes are pictured for ‘lost initial’ sectors (B, D) as well as ‘phloem only’ and ‘xylem only’ sectors (F, H).

Relative contributions of transgenic tissue were also measured for 50 PO and 103 XO sectors (expressed as %TG) and plotted in 10% classes (Fig. 4E–H). In the same way, size class distribution was again plotted for %TG in phloem and xylem, respectively, of LI sectors (Fig. 4A–D). In both XO and LI sectors, the frequency of %TG gradually decreases in xylem from small to high percentage classes, with a marked wave-like pattern for LI sectors. In contrast, PO sector size shows two clear frequency peaks for the 40–50% and 80% TG size classes, while phloem sector size in LI sectors only shows a single peak for the 70% TG class.

In summary, there seems to be no tight synchronization between phloem and xylem cell proliferation.

### Histological analyses

While macroscopic assessment provided an overview of developmental patterns, histological analyses were performed using higher resolution microscopy to reveal cellular details (Fig. 2). For this purpose, nine cambial sectors were analysed (one ‘lost initial’ and eight ‘full’ sectors). In all cases, GUS staining was found in xylem axial parenchyma in the dedifferentiation zone progressing outward from the wound parenchyma boundary often at sites with prolific wound tissue formation. Here, the tangential width of sectors was typically 6 (±1.1) cells but was observed to be as high as 11 and as low as 3. Macroscopic assessment of an additional 65 cambial sectors from a complementary trial showed that in 75% (49) of cases initial transformation had occurred in the area of higher wound tissue proliferation. Moving away from the wound site into the xylem, tangential sector width decreased on average by half (3 ± 0.6 cells) before widening at the cambium to on average 10 cells (±1.1) and further again in the phloem to a maximum average width of 13 (±1.8) cells at the point where phloem fibres were first discernible (note that GUS staining is strongest in parenchyma cells and decreases as cellular content is lost during the maturation of fibres and vessel elements). The width of cambial sectors then extended further toward the periderm at variable distances, but in most cases (six of the eight sectors) staining was observed up to the phloem/periderm boundary. In the ‘lost initial’ cambial sector, staining was absent across the cambium and in adjacent xylem and phloem cells (Fig. 2D, E).

Cell counts determined the average number of xylem derivatives created radially during the growth period in a ‘full’ cambial sector to be 119 (±11.9) with an average ratio of xylem cells in a radial file to sector width at the cambium of 13:1 (±2.2) where the highest ratio observed was 21:1 and the lowest 8:1. Radially, on average 33 (±3.6) phloem cells were created over this growth period with an average ratio of xylem to phloem derivatives of 4 (±0.5). Here, the highest ratio observed was 6:1 and the lowest of 2:1. The ‘lost initial’ cambial sector produced 60 transgenic and 13 non-transgenic xylem and 28 transgenic phloem and 9 non-transgenic phloem radially aligned derivatives over the growth period (Fig. 2D, E).

Histological analysis revealed the extent of tangential and radial expansion and thereby the rate of anticlinal and periclinal cell divisions of cambial initials and mother cells. It also visualizes cellular detail of the dedifferentiation zone where, following the initial wounding over a few cell cycles, a new cambium was established.

## Discussion

### Sector patterns, frequency, and origins

In total, 581 sectors were identified and assessed, largely conforming to previously described sector types (Spokevicius *et al.*, 2016; Van Beveren *et al.*, 2006) with few (eight) exceptions. Cells giving rise to cambial tissues, specifically cambial initials and mother cells, were by far the most susceptible to *Agrobacterium* transformation, accounting for >65% of all sectors identified. Cells giving rise to other sector types showed somewhat lower susceptibility, comprising 21% periderm, 9% wound parenchyma, and 5% tylose sectors. Although not quantified, there appeared to be a positive correlation between vigorous wound response and transformation efficiency in cambial tissue, suggesting that *Agrobacterium*-mediated transformation is more prevalent in rapidly dividing cells. These observations are consistent with previous studies that also showed transformation rates to be highest in cambial tissue with actively dividing cells (Van Beveren *et al.*, 2006).

### Not every transformed cell contributes to cambium formation

Not every transformed cell contributes to cambium formation. Transformed tyloses, for example, will be the result of transformation of vessel-associated ray parenchyma cells that in response to wounding penetrated into adjacent vessels to prevent air from entering the transpiration stream. Wound parenchyma sectors, too, most probably originated from transdifferentiation of ray and/or axial parenchyma cells within existing xylem forming wound parenchyma to seal the exposed wound site. These are clonally distinct from parenchyma cells in the dedifferentiation zone, as will be discussed below. Periderm sectors in turn will have originated from an originally transformed pre-existing periderm or advanced phloem cell. Since periderm cells within a bark flap are positioned at some distance from the exposed cambial tissue (and therefore are not directly affected by wounding), these will have been transformed following penetration of *Agrobacterium* solution through bark and other tissues via intercellular spaces. Periderm sectors at the edges of bark flaps that were found in wound-induced distorted tissues were excluded from analysis (see the Materials and methods).

### Wound closure occurs independently of cambium differentiation

New xylem exclusively formed on the outside of the wound parenchyma zone, indicating that when cambial windows were created, the cambium and/or cells that later would give rise to a new cambium remained with the bark flap. The fact that we did not find any sectors crossing the wound parenchyma/new xylem boundary or the phloem/periderm boundary provides evidence that neither wound parenchyma nor periderm cells contributed to the re-establishment of a cambium and, on the other hand, that pre-existing cambial cells did not contribute to any significant extent to wound closure. Instead, wound tissue will have primarily formed from live xylem cells (ray or axial parenchyma) found on the exposed xylem surface, while new xylem and phloem will have derived from reactivated cambial cells or other cells, leading to the formation of cambial cells in the bark flap. These findings are consistent with histological investigations into wound closure in a range of angiosperm and gymnosperm species (Sharples and Gunnery, 1933; Barker, 1954; Brown and Sax, 1962; Noel, 1968; Bangerter, 1984; Grosser *et al.*, 1991; Larson, 1994; Grunwald *et al.*, 2002; Van Beveren *et al.*, 2006), which demonstrated that the majority of the wound response occurs from ray parenchyma in the already differentiated xylem.

A corollary of our observations is that the proximal and distal boundaries of newly formed cambium-derived tissues are defined by the wound parenchyma/new xylem (dedifferentiation zone) border and, where present, the outward pointing border of transgenic phloem tissue, respectively.

### The poplar cambium features a single layer of stem cells

We base our analysis on the premises that (i) each sector originated from a single transformed cell, and therefore that (ii) common sector patterns are indicative of preferred developmental cell lineages.

Of the 379 sectors and sector patterns that are explained by transformation of cells within the cambial zone, 58% (221; Fig. 1A, E–G) can be traced to the transformation of a single cell that gave rise to a cambial initial, providing support for the notion that the preferred pattern of cambium organization in poplar is a single layer of cambial stem cells. Moreover, the existence of corresponding sectors in the phloem and xylem in LI and related sectors (LIX and LIP) provides evidence for frequent cell loss or invasive growth disrupting the lineage of a single originally transformed cambial initial (51 cases; Fig. 1E–G). Only in rare cases did we observe this type of disruption at the mother cell level (six cases for XMCs and two for PMCs; Fig. 1F, I and G, L, respectively). In contrast, if multiple layers of potential cambial initials would be present, their cell lineages would be equally likely to be disrupted by cell loss or invasive growth, resulting in split sectors that cross the cambium (Fig. 1B–D). No such patterns were observed in our experiments. With an average width of 10 cells, FC sectors also provide evidence of frequent anticlinal cell divisions within the initial cell layer, while LI as well as PO and XO sector widths are the result of anticlinal divisions at the respective mother cell levels (compare FC and LI sector examples in Fig. 2).

These findings resolve for poplar trees a debate that protracted over many decades. Observations from the cambium of some gymnosperms, often during dormancy when cell number in the cambial zone is at its lowest, suggested that the initiating layer is comprised of a single initial (Newman, 1956; Mahmood, 1968, 1990; Murmanis, 1970; Timell, 1980). These authors claimed to have identified the cambial initial by assessing past division events in newly formed xylem and phloem derivatives, in particular, by assessing the parental walls of these cells. During cell division, formation of a new primary wall around the daughter protoplasts occurs, leaving both daughter cells enclosed within a common wall derived from the parent cell. This concept is referred to as ‘emboxing’, and subsequent assessment of the parental cell walls of cells in radial files in the cambial zone was taken as evidence of its cell lineage (Mahmood, 1968, 1990; Timell 1980). For example, assessment of dormant cambia in *Picea abies* by Timell (1980) showed clear examples of the existence of Sanio’s four (see the Introduction) in some cells in the cambial zone, identifying the initial as the most centrifugal cell within a group of four ‘emboxed’ xylem derivatives. Other authors who have entered the debate in support of such a uniseriate model are Schmid (1976) and Bannan (1955, 1962) who argued along similar lines.

In contrast, support for a multiseriate model comes from Esau (1948) who was unable to identify an initial in the cambial zone of *Vitis*, an angiosperm species. Later, Catesson (1974, 1980, 1984) was also unable to identify initials positively within the cambium of the angiosperm *Acer pseudoplantanus*. In this work, Catesson assessed the frequency of anticlinal divisions within the cambial zone with the hypothesis that any cell showing greater mitotic activity was more likely to be an initiating cell. However, she observed similar rates of anticlinal divisions across the entire cambial zone and found new radial files created from anticlinal divisions of cells located at various positions within the cambial zone. In her studies and in support of a multiseriate model, Catesson also observed non-synchronous activity in the cambial zone leading to the offset of neighbouring initials, including the formation of gaps along the tangential plane between dividing cells. Similar concentrations of RNA were found in cells in the cambial zone, which were higher than those of more differentiated daughter cells and, most importantly, all cells found in the cambial zone had identical structural characteristics. Catesson concluded that several cells within a radial file in the cambium have the potential to be initials and cautioned against extending evidence gained in gymnosperms to angiosperms, suggesting that further, precise work on comparing these two phyla would be required before reaching general conclusions.

Placing a genetic label on individual cambial cells allowed us to trace their derivatives objectively, and the resulting patterns positively demonstrate a developmental preference for a single layer of cambial initials.

### Phloem and xylem differentiation are controlled independently

In our experiment, 51 of 221 patterns or 23% (Fig. 1E–G) of cambial sectors were described as ‘lost initial’ (LI) sector types, explained by the loss of a cambial initial and/or through cell invasion and subsequent intrusive growth. This indicates that approximately one in four cells that gave rise to a cambial initial lineage at the time of wounding was purged from the cambial zone across a growing season. However, the true extent of individual cambial initials being lost from the cambium is likely to be higher as the type of cell tagging used here is not informative about how many cambial initials were purged, just how many cambial cell lineages were lost.

Early or late loss of a cambial initial have opposite effects on subsequent phloem and XMC replenishment as indicated by the respective frequencies of LI sector patterns. Early loss of a single transformed cambial initial, for example, is indicated by a short xylem portion of this split sector type (indicating that an XMC was active only for a few cell cycles before being replenished) and these were observed significantly more frequently than split sectors with a longer (higher %TG) xylem portion (Fig. 4C). The opposite is true for the phloem portion of these sectors, with larger transgenic phloem portions being significantly more numerous than small ones (Fig. 4A). Accordingly, Fig. 4A–D depicts the pattern of cambial initial loss and/or replacement.

Simultaneously plotting %TG in phloem and xylem of LI sectors shows that as a general trend smaller xylem portions align with smaller phloem portions in individual LI sectors; however, any phloem size class can be aligned with almost every xylem size class, indicating that phloem and xylem differentiation are not synchronized and are therefore likely to be controlled independently (Fig. 3).

These results are largely consistent with observations by Bannan (1950, 1965, 1968), Cumbie (1969), and Evert (1961) who reported that the number of anticlinal divisions occurring in fusiform initials is often higher than required to achieve the measured increase in stem circumference. Superfluous cells are either lost or squeezed out from the cambial zone or undergo transdifferentiation to form ray initials. For example, in *Chamaecyparis*, a conifer tree species, only eight new fusiform initials were maintained within a defined region of the cambial zone after a total of 65 anticlinal divisions, while in a further two defined regions two remained after 26 and six remained after 36 divisions (Bannan, 1950). Attempts to apply mathematical models to the division of cells in the cambial zone in *Populus* by Barlow *et al.* (2002) have also shown that the actual number of cells in the cambial zone is often higher than is required to compensate for circumference expansion.

### Phloem and xylem mother cells

While full cambial sectors shed light on both periclinal and anticlinal division patterns and frequency of cambial initials, phloem and xylem only sectors as well as LI sectors allow us to determine the contribution of mother cells to radial and tangential expansion. This is based on the premise that PO and XO sectors result from transformation of a single mother cell rather than a cambial initial and that LI sector separation occurs after a single transformed initial divides periclinally (at least once in the case of cell invasion or at least twice in the case of cell loss) and not yet anticlinally. Also in this case, subsequent lateral expansion is the result of anticlinal divisions in mother and potentially daughter cells (data not shown; see example in Fig. 2D, E). Cell loss or replacement of mother and daughter cells is rare but possible, as evidenced by the infrequent observation of LIX, LIP, SX, and SP sectors (Fig. 1F, G, I, L).

### Patterns of replenishment differ between phloem and xylem mother cells

Periclinal or additive divisions account for ~90% of cell divisions in the cambial zone, leading to the formation of highly organized files of radially aligned cells (Berlyn, 1982; Iqbal and Ghouse, 1990; Lachaud *et al.*, 1999). Typically, during normal stem growth, XMCs are mitotically more active than PMCs, leading to a disproportionate ratio of xylem to phloem elements, although this varies between species and physiological state. In our experiments, we observed an average ratio of four xylem to one phloem derivative which aligns well with observations by Waisel *et al.* (1966) in *Eucalyptus camaldulensis* who reported that approximately four xylem derivatives were produced for every one phloem derivative regardless of changes in environmental conditions including photoperiod, thermoperiod, and season. In *Populus euramericana*, Stahel (1968) observed a ratio of 10:1 during normal growth conditions, and Larson (1994) in a review of this subject presented a selection of xylem/phloem ratios from several tree species and found values that ranged between 15:1 and 1:1. He also noted that in most cases increased growth rates led to increases in the number of divisions in XMCs.

If our assumption is correct that PO and XO sectors derived from a single transformed PMC or XMC, respectively, then the radial lengths of resulting sectors are indicative of the time for which these mother cells remained active or the percentage contribution they made to radial expansion before being replenished by a new mother cell contributed by periclinal division of a cambial initial. These %TG contributions to radial phloem or xylem production are plotted for 10% size classes in Fig. 4E–H. One observation is that the period for which mother cells remain active varies greatly and that therefore the time or the number of cell cycles for which a mother cell remains active is not pre-determined. Supporting this notion are observations of full cambial sectors where the ratio of periclinal to anticlinal derivatives ranges from as low as 8:1 to as high as 21:1; however, as these figures only relate to transformed sectors, these ratios are likely to be an upper estimate.

While relative contributions by PMC and XMC are not pre-determined, there are clear patterns emerging as the percentage contributions by PMCs show two peaks in observed frequency at 40% and 80% contribution to newly formed phloem (Fig. 4E) while the frequency of percentage contributions by XMCs steadily declines from 10% to 90% contribution classes in newly formed xylem (Fig. 4G). If the extent to which individual mother cells contribute to phloem or xylem production is not pre-determined, their eventual replenishment through co-ordinated division of cambial initials must be the result of fine-tuned cell to cell communication most probably via hormonal or molecular cues (Aloni *et al.*, 2000; Dengler, 2001; Savidge, 2001; Fukuda, 2004; Zhang *et al.*, 2015, and many more).

### Conclusions

By taking a step back and mainly looking at patterns rather than cellular detail, our studies provide new insights into cambium dynamics and cambial cell identity. In doing so, we showed that the poplar cambium most probably features a single layer of true cambial initials, that initials are frequently lost from the cambium, that such cell loss rarely occurs at the mother cell level, that phloem and xylem differentiation are controlled independently, and that the frequency of mother cell replenishment is not pre-determined. Largely focusing on radial cambium differentiation with some limited observations on lateral expansion, we did not attempt to investigate developmental patterns in the longitudinal direction and neither did we investigate distinct developmental patterns for ray versus fusiform initials.

Just like other cell and tissue culture methods that could be used for the study of cambium differentiation and wood formation, the present somatic sector analysis has its limitations, in particular because an initial wound response is unavoidable and, when relying on GUS assays, destructive sampling is required to identify transgenic sectors (for a review, see Spokevicius *et al.*, 2007). Wounding could be avoided by including mobile genetic elements in our gene constructs for the creation of variegated whole transgenic plants, but detection of informative transgenic tissue sectors might prove difficult. Development and use of suitable fluorescent marker genes on the other hand in combination with confocal microscopy would allow for non-destructive assessment of samples.

Nonetheless, and despite the listed limitations, here we were able to provide a developmental baseline for future studies investigating if and how modified gene expression, in particular expression of transcription factors, might affect overall patterns of cambium differentiation and/or their respective frequencies.

## Supplementary data

Supplementary data are available at *JXB* online.

Table S1. A comprehensive table of samples and identified sectors.

Supplementary Table S1

## References

[CIT0001] Aloni R, Feigenbaum P, Kalev N, Rozovsky S 2000 Hormonal control of vascular differentiation in plants: the physiological basis of cambium ontogeny and xylem evolution. In: Savidge RA, Barnett JR, Napier R, eds. Cell and molecular biology of wood formation. Oxford: BIOS Scientific Publishers Ltd, 223–236.

[CIT0002] Bailey IW 1923 The cambium and its derivative tissues IV: the increase in girth of the cambium. American Journal of Botany 10, 499–509.

[CIT0003] Bangerter UM 1984 Der Verschlussmechanismus von Längswunden am Stamm von *Larix decidua* Mill. und *Picea abies* (L.) Karst. Vierteljahrsschrift der Naturforschenden Gesellschaft in Zürich 129, 339–398.

[CIT0004] Bannan MW 1950 The frequency of anticlinal divisions in fusiform cambial cells of *Chamaecyparis*. American Journal of Botany 37, 511–519.

[CIT0005] Bannan MW 1955 The vascular cambium and radial growth in *Thuja occidentalis* L. Canadian Journal of Botany-Revue Canadienne De Botanique 33, 113–138.

[CIT0006] Bannan MW 1962 The vascular cambium and tree-ring development. In: Koslowski TT, ed. Tree growth. New York: The Ronald Press, 3–21.

[CIT0007] Bannan MW 1965 Ray contacts and rate of anticlinal division in fusiform cambial cells of some *Pinaceae*. Canadian Journal of Botany-Revue Canadienne De Botanique 43, 487–508.

[CIT0008] Bannan MW 1968 Anticlinal divisions and organization of conifer cambium. Botanical Gazette 129, 107–113.

[CIT0009] Barker WG 1954 A contribution to the concept of wound repair in woody stems. Canadian Journal of Botany-Revue Canadienne De Botanique 32, 486–490.

[CIT0010] Barlow PW, Brain P, Powers SJ 2002 Estimation of directional division frequencies in vascular cambium and in marginal meristematic cells of plants. Cell Proliferation 35, 49–68. 11856178 10.1046/j.1365-2184.2002.00225.x PMC6734916

[CIT0011] Berlyn GP 1982 Morphogenic factors in wood formation and differentiation. In: Baas P, ed. New perspectives in wood anatomy. The Hague/Boston/London: Martinus Nijhoff/Dr W Junk Publishers, 123–150.

[CIT0012] Bossinger G, Smyth DR 1996 Initiation patterns of flower and floral organ development in *Arabidopsis thaliana*. Development 122, 1093–1102. 8620836 10.1242/dev.122.4.1093

[CIT0013] Bossinger G, Spokevicius AV 2011 Plant chimaeras and mosaics. In: Encyclopedia of life sciences. Chichester: John Wiley & Sons, Ltd.

[CIT0014] Brown CL, Sax K 1962 Influence of pressure on differentiation of secondary tissues. American Journal of Botany 49, 683–691.

[CIT0015] Butterfield BG 1975 Terminology used for describing the cambium. IAWA Bulletin 1, 13–14.

[CIT0016] Catesson AM 1974 Cambial cells. In: Robards AW, ed. Dynamic aspects of plant ultrastructure. London: McGraw-Hill Book Co, 358–390.

[CIT0017] Catesson AM 1980 The vascular cambium. Control of shoot growth in trees. In: Little CHA, ed. Proceedings of the joint workshop of IUFRO working parties on xylem and shoot growth physiology. New Brunswick, Canada: IUFRO, 12–40.

[CIT0018] Catesson AM 1984 Cambial dynamics. Annales des Sciences Naturelles-Botanique et Biologie Vegetale 6, 23–43.

[CIT0019] Catesson AM, Lachaud S 1993 The cambium, structure, function and control of its seasonal activity. Acta Botanica Gallica 140, 337–350.

[CIT0020] Cumbie BG 1969 Developmental changes in vascular cambium of *Polygonum lapathifolium*. American Journal of Botany 56, 139–146.

[CIT0021] Dengler NG 2001 Regulation of vascular development. Journal of Plant Growth Regulation 20, 1–13.

[CIT0022] Esau K 1948 Phloem structure in the grapevine, and its seasonal changes. Hilgardia 18, 217–296.

[CIT0023] Etchells JP, Mishra LS, Kumar M, Campbell L, Turner SR 2015 Wood formation in trees is increased by manipulating PXY-regulated cell division. Current Biology 25, 1050–1055. 25866390 10.1016/j.cub.2015.02.023 PMC4406943

[CIT0024] Etchells JP, Provost CM, Mishra L, Turner SR 2013 *WOX4* and *WOX14* act downstream of the PXY receptor kinase to regulate plant vascular proliferation independently of any role in vascular organisation. Development 140, 2224–2234. 23578929 10.1242/dev.091314 PMC3912870

[CIT0025] Evert RF 1961 Some aspects of cambial development in *Pyrus communis*. American Journal of Botany 48, 479–488.

[CIT0026] Fisher K, Turner S 2007 PXY, a receptor-like kinase essential for maintaining polarity during plant vascular-tissue development. Current Biology 17, 1061–1066. 17570668 10.1016/j.cub.2007.05.049

[CIT0027] Fukuda H 2004 Signals that control plant vascular cell differentiation. Nature Reviews. Molecular Cell Biology 5, 379–391. 15122351 10.1038/nrm1364

[CIT0028] Grosser D, Lesnino G, Schulz H 1991 Histologic investigations of the protection wood of indigenous hardwoods. Holz als Roh-und Werkstoff 49, 65–73.

[CIT0029] Grunwald C, Stobbe H, Schmitt U 2002 Developmental stages of callus formation on wound edges of broad-leaved trees. Forstwissenschaftliches Centralblatt 121, 50–58.

[CIT0030] Hirakawa Y, Kondo Y, Fukuda H 2010 TDIF peptide signaling regulates vascular stem cell proliferation via the *WOX4* homeobox gene in *Arabidopsis*. The Plant Cell 22, 2618–2629. 20729381 10.1105/tpc.110.076083 PMC2947162

[CIT0031] Iqbal M, Ghouse AKM 1990 Cambial concept and organisation. In: Iqbal M, ed. The vascular cambium. Taunton, UK: Research Studies Press, 1–36.

[CIT0032] Kilby NJ, Fyvie MJ, Sessions RA, Davies GJ, Murray JA 2000 Controlled induction of GUS marked clonal sectors in *Arabidopsis*. Journal of Experimental Botany 51, 853–863. 10948211

[CIT0033] Köhl M, Lasco R, Cifuentes M, et al 2015 Changes in forest production, biomass and carbon: results from the 2015 UN FAO global forest resource assessment. Forest Ecology and Management 352, 21–34.

[CIT0034] Kondo Y, Ito T, Nakagami H, Hirakawa Y, Saito M, Tamaki T, Shirasu K, Fukuda H 2014 Plant GSK3 proteins regulate xylem cell differentiation downstream of TDIF–TDR signalling. Nature Communications 5, 3504. 10.1038/ncomms4504 24662460

[CIT0035] Lachaud S, Catesson AM, Bonnemain JL 1999 Structure and functions of the vascular cambium. Comptes rendus de l’Academie des sciences. Serie III, Sciences de la vie 322, 633–650. 10.1016/s0764-4469(99)80103-6 10505236

[CIT0036] Larson PR 1994 The vascular cambium. New York, Berlin, Heidelberg: Springer-Verlag.

[CIT0037] Lazo GR, Stein PA, Ludwig RA 1991 A DNA transformation-competent *Arabidopsis* genomic library in *Agrobacterium*. Bio/Technology 9, 963–967. 1368724 10.1038/nbt1091-963

[CIT0038] Lev-Yadun S, Aloni R 1995 Differentiation of the ray system in woody-plants. Botanical Review 61, 45–84.

[CIT0039] Mahmood A 1968 Cell grouping and primary wall generations in cambial zone xylem and phloem in *Pinus*. Australian Journal of Botany 16, 177–195.

[CIT0040] Mahmood A 1990 The parental cell wall. In: Iqbal M, ed. The vascular cambium. Taunton, UK: Research Studies Press, 113–125.

[CIT0041] Murmanis L 1970 Locating the initial in vascular cambium of *Pinus strobus* by electron microscopy. Wood Science and Technology 4, 1–14.

[CIT0042] Nesbitt MN, Gartler SM 1971 The applications of genetic mosaicism to developmental problems. Annual Review of Genetics 5, 143–162. 10.1146/annurev.ge.05.120171.001043 16097654

[CIT0043] Newman IV 1956 Pattern in the meristem of vascular plants. I. Cell partition in living apices and in the cambial zone in relation to concepts of initial cells and apical cells. Phytomorphology 6, 1–19.

[CIT0044] Noel ARA 1968 Callus formation and differentiation at an exposed cambial surface. Annals of Botany 32, 347–359.

[CIT0045] Pan Y, Birdsey RA, Fang J, et al 2011 A large and persistent carbon sink in the world’s forests. Science 333, 988–993. 21764754 10.1126/science.1201609

[CIT0046] Raatz W 1892 Die Stabbildungen im secondären Holzkörper der Bäume und die Initialentheorie. Jahrbücher für Wissenschaftliche Botanik 23, 567–636.

[CIT0047] Sanio K 1873 Anatomie der gemeinen Kiefer (*Pinus sylvestris* L.). Jahrbücher für Wissenschaftliche Botanik 9, 50–126.

[CIT0048] Saulsberry A, Martin PR, O’Brien T, Sieburth LE, Pickett FB 2002 The induced sector *Arabidopsis* apical embryonic fate map. Development 129, 3403–3410. 12091310 10.1242/dev.129.14.3403

[CIT0049] Savidge RA 2001 Intrinsic regulation of cambial growth. Journal of Plant Growth Regulation 20, 52–77.

[CIT0050] Scheres B, Wolkenfelt H, Willemsen V, et al 1994 Embryonic origin of the *Arabidopsis* primary root and root-meristem initials. Development 120, 2475–2487.

[CIT0051] Schmid R 1976 The elusive cambium—another terminological contribution. IAWA Bulletin 4, 51–59.

[CIT0052] Sharples A, Gunnery H 1933 Callus formation in *Hibiscus rosa-sinensis* L and *Hevea brasiliensis* mull. arg. Annals of Botany 47, 827–839.

[CIT0053] Spena A, Salamini F 1995 Genetic tagging of cells and cell layers for studies of plant development. Methods in Cell Biology 49, 331–354. 8531767 10.1016/s0091-679x(08)61464-8

[CIT0054] Spokevicius AV, Taylor L, Melder E, et al 2016 The use of induced somatic sector analysis (ISSA) for studying genes and promoters involved in wood formation and secondary stem development. Journal of Visualized Experiments 116, e54553. 10.3791/54553 PMC5092166 27768077

[CIT0055] Spokevicius AV, Tibbits JFG, Bossinger G 2007 Whole plant and plant part transgenic approaches in the study of wood formation—benefits and limitations. Transgenic Plant Journal 1, 49–59.

[CIT0056] Spokevicius AV, Van Beveren KS, Bossinger G 2006 *Agrobacterium*-mediated transformation of dormant lateral buds in poplar trees reveals developmental patterns in secondary stem tissues. Functional Plant Biology 33, 133–139. 10.1071/FP05176 32689220

[CIT0057] Stahel J 1968 Quantitative und qualitative Alterungsphänomene in Pappeln (*Populus × euramericana* (Dode) Guinier cv. ‘Robusta’. Holz als Roh-und Werkstoff 26, 418–427.

[CIT0058] Szymkowiak EJ, Sussex IM 1996 What chimeras can tell us about plant development. Annual Review of Plant Physiology and Plant Molecular Biology 47, 351–376. 10.1146/annurev.arplant.47.1.351 15012293

[CIT0059] Timell TE 1980 Organization and ultrastructure of the dormant cambial zone in compression wood of *Picea abies*. Wood Science and Technology 14, 161–179.

[CIT0060] Van Beveren KS, Spokevicius AV, Tibbits JG, Wang Q, Bossinger G 2006 Transformation of cambial tissue *in vivo* provides an efficient means for induced somatic sector analysis and gene testing in stems of woody plant species. Functional Plant Biology 33, 629–638. 10.1071/FP06057 32689272

[CIT0061] Waisel Y, Noah I, Fahn A 1966 Cambial activity in *Eucalyptus camaldulensis* Dehn. 2. Production of phloem and xylem elements. New Phytologist 65, 319–324.

[CIT0062] Zhang J, Alonso Serra JA, Helariutta Y 2015 Wood development: growth through knowledge. Nature Plants 1, 15060.

